# Terminal Schwann cell and vacant site mediated synapse elimination at developing neuromuscular junctions

**DOI:** 10.1038/s41598-019-55017-w

**Published:** 2019-12-09

**Authors:** Jae Hoon Jung, Ian Smith, Michelle Mikesh

**Affiliations:** 1 Department of Biology, Texas A&M University, College Station, TX 77843 USA; 2 Laboratory of Neurobiology, National Institute of Neurological Disorders and Stroke, National Institutes of Health, Bethesda, MD 20892 USA; 3 Institute for Neuroscience, Texas A&M University, College Station, TX 77843 USA; 4 Section of Molecular Cell and Developmental Biology, School of Biological Sciences, Institute of Cell and Molecular Biology and Neuroscience, University of Texas at Austin, Austin, TX 78712 USA

**Keywords:** Synaptic development, Biophysical models, Scanning electron microscopy

## Abstract

Synapses undergo transition from polyinnervation by multiple axons to single innervation a few weeks after birth. Synaptic activity of axons and interaxonal competition are thought to drive this developmental synapse elimination and tested as key parameters in quantitative models for further understanding. Recent studies of muscle synapses (endplates) show that there are also terminal Schwann cells (tSCs), glial cells associated with motor neurons and their functions, and vacant sites (or vacancies) devoid of tSCs and axons proposing tSCs as key effectors of synapse elimination. However, there is no quantitative model that considers roles of tSCs including vacancies. Here we develop a stochastic model of tSC and vacancy mediated synapse elimination. It employs their areas on individual endplates quantified by electron microscopy-based analyses assuming that vacancies form randomly and are taken over by adjacent axons or tSCs. The model reliably reproduced synapse elimination whereas equal or random probability models, similar to classical interaxonal competition models, did not. Furthermore, the model showed that synapse elimination is accelerated by enhanced synaptic activity of one axon and also by increased areas of vacancies and tSCs suggesting that the areas are important structural correlates of the rate of synapse elimination.

## Introduction

The vertebrate neuromuscular junction (NMJ), a synapse between a motor neuron and a muscle fiber, has been intensely studied to understand general synaptic function due to its accessibility and relatively large size compared to other synapses found in the nervous system^1,2^. Decades of study has expanded our understanding of this synapse and how its components work together: the presynaptic motor axon and its nerve terminal, the postsynaptic muscle fiber and its specialized synaptic region, the endplate, and the non-myelinating terminal Schwann cell (tSC), the glia of the peripheral motor synapse. In a mature NMJ, a single presynaptic nerve terminal releases the neurotransmitter acetylcholine to the endplate where they bind acetylcholine receptors and depolarize the muscle fiber resulting in contraction. However, at birth each endplate is contacted by an excess of nerve terminals and those extra nerve terminals must be removed to ensure proper synaptic function. Thus, the removal of these superfluous nerve inputs, or synapse elimination, is a critical step in the maturation not just of the synapses themselves but also the overall development of the nervous system during the first few weeks of life^3–12^.

Synapse elimination is well characterized as a competitive process of different axons by both experimental and modeling studies^3,4,11,13,14^. At dually innervated mouse NMJs, one axon was observed to expand its territory as the other retracted^11^. The random competition among different axons was successfully shown to simulate removal of extra axonal connections in an endplate^8^. Several other models largely based on interaxonal competition were constructed to match behaviors of synapse elimination^9,10,15–24^. Though interaxonal competition undoubtedly plays a significant role in the progression of each developing synapse to a mature, singly-innervated form, there is growing evidence that Schwann cells are also important in synapse elimination^7,25–28^. Schwann cells, tightly associated with motor neurons and their functions, have been observed removing retracting motor axons^25,28^. A recent serial electron microscope study of developing mouse NMJs showed that endplates are covered with multiple axons, tSCs, and vacant sites (or vacancies) that have no such contact from either axons or tSCs; the study suggested that tSCs promote postnatal synapse elimination not only by phagocytosis of retracting motor neurons but also by direct competition with motor neurons for occupation of the maturing endplate^7^. A subsequent study showed that overexpression of neuregulin1 type 3 (NRG1-III), a potent activator of tSCs, led to expedited synapse elimination and that by reducing the activity of endogenous NRG1-III, single-innervation is achieved more slowly than control littermates^26^. The findings support a direct role of tSCs in synapse elimination and provided a qualitative model of tSC-mediated synapse elimination. However, there is no quantitative model of synapse elimination that incorporates this role despite several models considering dynamic competition of different axons^8–10,15–24^, and the possible contribution of the observed vacancies to synapse elimination is little understood.

With this in mind, we developed a quantitative model of synapse elimination that, for the first time, employs the contact areas of tSCs and vacancies in endplates to further understand the roles of tSCs and vacancies in synapse elimination. Using data from our previous studies^7,26^ and other newly obtained data for this study (See Methods), the transition probabilities and interactions among tSCs, vacancies, and axons were quantified as the key parameters of the model using Markov chain methods^29–35^. Our model assumed that a randomly formed vacancy can be taken over by adjacent axons or tSCs allowing the competition among different axons and also between tSCs and axons and that the probabilities of their taking over is directly related with their relative areas in an endplate. Our model simulated synapse elimination reliably, and the simulation results showed the coexistence of tSCs, vacancies, and axons in endplates throughout the entire process of synapse elimination consistent with experimental observations of developing mouse NMJs^7^. In contrast, when tSCs, vacancies, and axons were assumed to compete with random or equal transition probabilities similar to other models that do not consider the competition of tSCs and vacancies^36^, synapse elimination proceeded unreliably. Our simulation results also showed that the relative area of the vacancy or tSC is positively correlated with the rate of synapse elimination. Furthermore, our results exhibited acceleration and delay of synapse elimination depending on degrees of synaptic activity of axons, which are consistent with other studies and recent observations^26,27^. Our findings show that our model produces results supporting the active role of tSCs in synapse elimination and also suggesting the direct relationship of the relative areas of tSCs and vacancies with the rate of synapse elimination. Our model and its predicted results may contribute to discovering structural correlates of synapse elimination providing a more complete framework for further understanding early synapse development.

## Results

### Motor neurons, terminal Schwann cells (tSCs), and vacant sites (vacancies) present on an endplate of a muscle fiber at the day of birth (P0), the third postnatal day (P3), the seventh postnatal day (P7), and the sixteenth postnatal day (P16)

**Figure 1:**
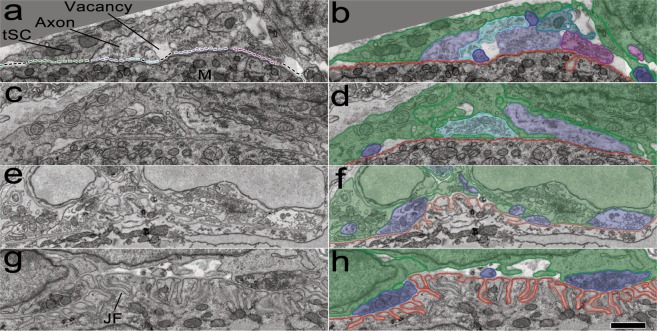
Developing sternomastoid NMJs at P0, P3, P7, and P16. (**a,c,e,g**) Montaged 2D electron micrographs spanning the entire NMJs in individual thin sections of mouse sternomastoid muscle fibers at P0, P3, P7, and P16, respectively. The nerve terminals of the NMJs contain synaptic vesicles and come within ~50 nm of the surface of a muscle fiber (M). The endplate on the muscle fiber is occupied by  tSCs (green dotted line), vacancies (black dotted line), and axons (dotted line in color different from green and black). Note that the postsynaptic membranes at P0 and P3 are smooth but show multiple indentations called junctional folds (JFs) at P7 and P16. Multiple axons at the NMJs are also associated with tSCs; vacancies are present, some of which extend to the surfaces of the muscle fibers. (**b,d,f,h**) Segmentation of the muscle fiber surfaces (red), terminals of axons (here in shades of blue), tSCs (green), and vacancies at the same NMJs. Red line represents the muscle fiber surface. Scale bar, 1 µm.

Serial electron micrographs of multiple NMJs in sternomastoid muscles at P0, P3, P7, and P16 (Fig. 1) were examined. The micrographs of 20 NMJs in the muscles at P0, P3, and P16 obtained by serial-section electron microscopy from a previous study^7^ were used while those of 5 NMJs in the muscles at P7 were newly obtained by serial block-face scanning electron microscopy and examined for this study. The images in Fig. 1a,c,e,g show that each endplate is occupied by multiple axons, tSCs, and vacancies. Axon nerve terminals (purple, cyan and blue in Fig. 1) are outlined in purple, and tSCs surrounding the axons are present at all endplates (color-coded in green in Fig. 1b,d,f,h). Vacant sites (vacancies) devoid of contacts of either tSCs or axons are marked by black lines (Fig. 1a). The postsynaptic membranes of the muscle fibers are outlined in red and exhibit junctional folds (JFs) by P7, a feature of normal development and mature NMJs.

**Figure 2:**
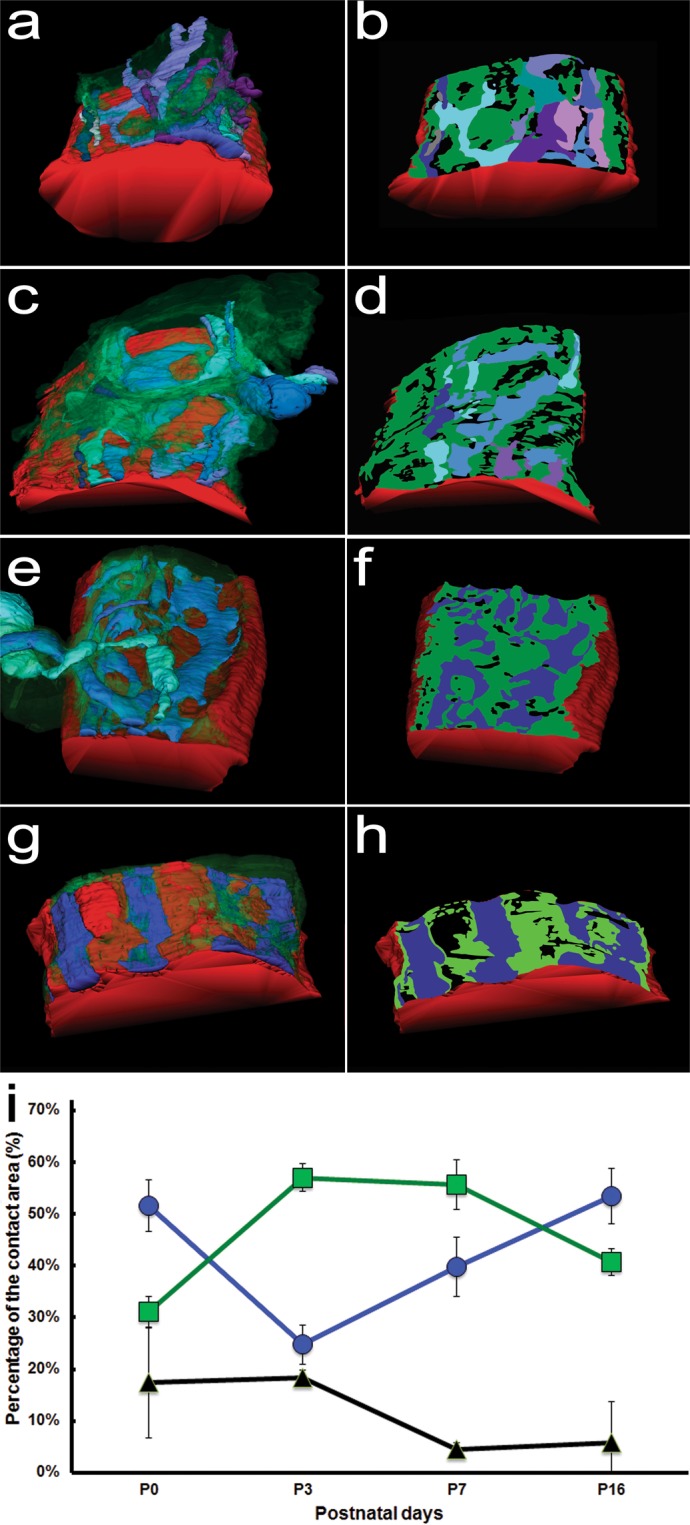
Surface models of axons of different neurons, tSCs, and vacancies at the NMJs and their contact areas on their endplate. (**a,c,e,g**) Surface rendering of a series of montaged electron micrographs of the sections of the same NMJs at P0, P3, P7, and P16, respectively. Axons, tSCs, and vacancies are color-coded in the same with Fig. 1. (**b,d,f,h**) The regions of the axons, tSCs, and vacancies in proximity to their muscle fiber or footprints at their endplate were segmented and using the series of their segmented lines the contact regions of the axons, tSCs, and vacancies were marked in color using the same color-code on their muscle fiber (red). (**i**) The average relative areas at P0, P3, P7, and P16. From P0 to P3, the relative contact area of tSCs in the endplate increased from 31.0% to 57.0% on average whereas that of axons decreased from 51.6% to 24.8% on average and also that the relative area of vacancies changed little (17.4% at P0 and 18.3% at P3 on average). The relative contact area of tSCs changed little from 57.0% at P3 to 55.6% at P7 and then decreased to 40.7% at P16. The relative contact area of vacancies markedly decreased from 18.3% at P3 to 4.55% at P7 and then changed little (5.82% at P16) whereas the relative contact area of axons significantly increased from 24.8% at P3 to 39.8% at P7 and then to 53.5% at P16.

The three-dimensional (3D) surface models of the tSCs (green), vacancies (black), and axons (other colors) were generated after they were traced or segmented (Fig. 2a–h) as in the previous study^7^. The sites of the tSCs, axons, and vacancies opposed to their endplate were identified (See Fig. 1a) and their contact areas were measured by computing the entire area of each contact site using their surface models generated after segmentation (See Methods). The measured areas were normalized by dividing the areas by the total areas of tSCs, vacancies, and axons to compare their relative contact areas. From P0 to P3, the relative contact area of tSCs in the endplate increased on average whereas that of axons decreased and the relative area of vacancies changed little (Fig. 2i) as seen in the previous study^7^. We also found that the relative contact area of tSCs changed little between P0 and P3, and then decreased (Fig. 2i). The dominance of tSC occupation from P3 to P7 is consistent with its active role in synapse elimination by competing against axons to occupy the territory of the endplate proposed in the previous study^7^. In contrast, the relative contact area of vacancies was found to markedly decrease from P3 to P7 and then change little by P16, whereas the relative contact area of axons significantly increased from P3 to P16 (Fig. 2i). The increase in the axonal area suggests that axons pursue territory of the endplate more actively than tSCs and vacancies. Considering the involvement of Schwann cells and axons in the competition^7,26^, these dynamic changes are expected to be closely related to the process of synapse elimination. However, even with current advanced imaging technologies, direct observation of these dynamic changes is extremely challenging to quantify. Accordingly, we set out to take a modeling approach to investigate the relationship of the dynamic change in the relative areas of tSCs, vacancies, and axons within the endplate during synapse elimination.

### A model of synapse elimination involving direct roles of tSCs and vacancies

**Figure 3:**
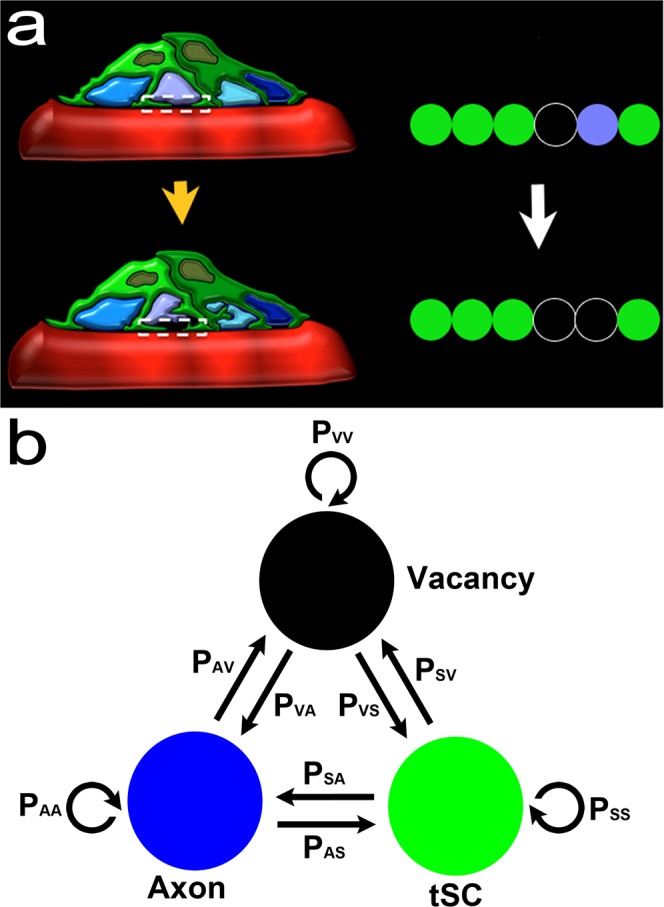
Schematic diagram of competing tSCs, vacancies, and axons. (**a**) An endplate covered with four different types of axon terminals (light blue, purple, cyan, and dark blue) and a tSC (green) transitions into an endplate covered with three of the axon terminals after removal of one axon terminal from the four and a vacancy (black) that takes the  place of the removed axon. (**b**) Competition of tSCs, vacancies, and axons. A blue circle represents a synaptic site formed on a muscle fiber by an axon. A green circle represents a synaptic site formed on a muscle fiber by a terminal Schwann cell (tSC). A black circle represents a vacancy having no axon or tSC on a muscle fiber. All the three different synaptic sites can transition from one kind of synaptic sites to another. A contact site formed by an axon has three different transition probabilities: a probability of transitioning into a vacancy (P_AV_), a probability of transitioning into a tSC (P_AS_), and a probability of no transition (P_AA_ = 1-P_AV_-P_AS_). Similarly, a contact site formed by a tSC has three different transition probabilities: a probability of transitioning into a vacancy (P_SV_), a probability of transitioning into an axon (P_SA_), and a probability of no transition (P_SS_ = 1−P_SA_−P_SV_). A vacant site also has three different transition probabilities: a probability of transitioning into a tSC (P_VS_), a probability of transitioning into an axon (P_VA_), and a probability of no transition (P_VV_ = 1−P_VS_−P_VA_).

**Figure 4:**
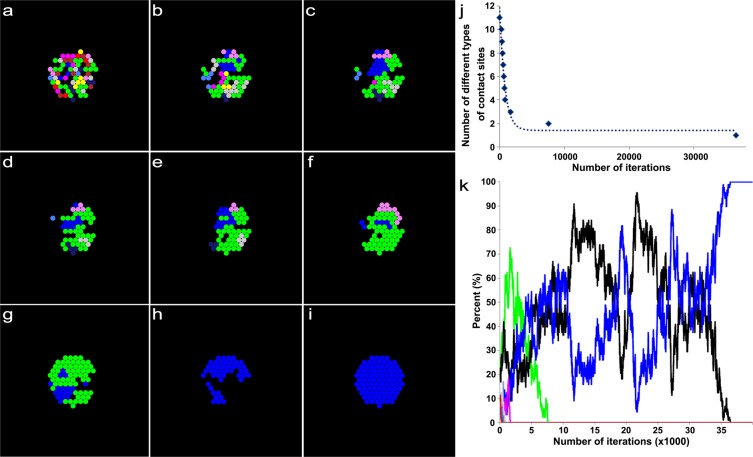
An example result based on a model of synaptic competition among axons, tSCs, and vacancies with random transition probabilities. (**a**) Initially, 9 different axons, tSCs and vacancies form their contact sites randomly in an endplate on a muscle fiber. The initial ratio of their total contact areas (axons:tSCs:vacancies) is about 30:16:54, which was determined from a previous serial electron microscopy study on developing muscle fibers of mouse during synapse elimination (See Methods). (**b–h**) The competition among axons, tSCs, and vacancies with their random transition probabilities shows elimination of multiple contact sites formed by different axons, tSCs, and vacancies at different iterations of the simulation (400, 500, 600, 700, 800, 1700, and 7600 iterations, respectively). (**i**) When the simulation reached 36500, synapse elimination is complete. However, only one type of axonal sites remains with no tSC and vacant sites that are present during and after synapse elimination of developing neuromuscular junctions. (**j**) The number of different types of contact sites in the endplate reduces sharply down to one as the iteration proceeds. (**k**) Change in the ratios of the contact areas formed by tSCs (green), vacancies (black), and 9 different axons (colors different from green and black) as the simulation based on a model of synaptic competition among axons, tSCs, and vacancies with random transition probabilities proceeds.

**Figure 5:**
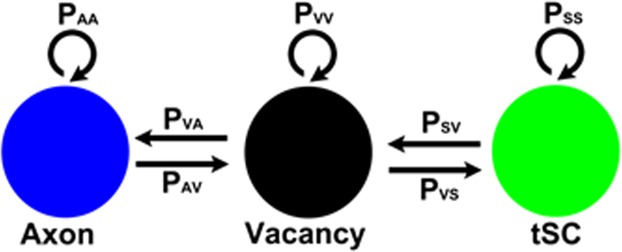
Schematic diagram of vacancy mediated competition between tSCs and axons. A model of competition between axonal and tSC sites assumes that tSC-axon competition to occupy the territory of the endplate is mediated by their adjacent vacancies. Accordingly, a synaptic site formed by an axon has two different transition probabilities: the probability of transitioning from an axonal site into a vacancy (P_AV_) and a probability of no transition (P_AA_ = 1−P_AV_). Similarly, a synaptic contact site formed by a tSC has two different transition probabilities: a probability of transitioning into a vacancy (P_SV_) and a probability of no transition (P_SS_=1−P_SV_); a vacant site that are not an axonal site either a tSC site has two different transition probabilities: a probability of transitioning into an axon (P_VA_) and a probability of transitioning into a tSC (P_VS_ = 1−P_VA_).

Previous modeling studies have shown the transition of a polyinnervated synapse to a singly innervated synapse is a result of competition among different axons in an endplate^8,36^. However, the models did not address the presence of tSCs and vacancies at endplates^7^. To address their roles, we simplified the contact sites of tSCs, vacancies, and axons in each endplate so that the transition of the endplate is represented as the reorganization of their simplified contact sites (Fig. 3a), and based on the previous studies^7,26^, we assumed that tSCs, vacancies, and axons compete against each other (Fig. 3b). Then a synaptic site formed by an axon has three different transition probabilities: a probability of transitioning into a vacancy (P_AV_), a probability of transitioning into a tSC (P_AS_), and a probability of no transition (P_AA_ = 1-P_AV_-P_AS_). Similarly, a synaptic contact site formed by a tSC has three different transition probabilities: a probability of transitioning into a vacancy (P_SV_), an axon (P_SA_), and no transition (P_SS_ = 1−P_SV_−P_SA_). A vacant site also has three different transition probabilities: a probability of transitioning into a tSC (P_VS_), an axon (P_VA_), and no transition (P_VV_ = 1−P_VS_−P_VA_). Therefore, there are six independent transition probabilities (P_AV_, P_AS_, P_SA_, P_SV_, P_VA_, and P_VS_); however, measuring such transition probabilities experimentally with sufficient observations is currently impossible. So we took a computational modeling approach here. We first constructed the initial layout of the endplate composed of multiple contact sites made by tSCs (green), vacancies (black), and axons (other colors) using the empirical data of synaptic footprints gathered from serial images of mouse sternomastoid muscles at P0 (See Figs. 1 and 2). These initial layouts, as seen in Fig. 4a,f,k, depict the relative contact areas of the sites of tSCs, vacancies, and axons measured in the P0 data sets and the random distribution of those sites. As noted above, it is reasonable to assume that those sites compete to take over other sites in the endplate. However, unlike axonal competition models of synapse elimination^8^ if those individual sites interact with each other with random transition probabilities, synapse elimination would proceed unreliably because it cannot account for the maintained presence of tSCs and vacancies after synapse elimination is complete. To test this possibility, we generated an idealized endplate randomly occupied by multiple contact sites of tSCs, vacancies, and axons. We kept the area ratios nearly those measured at P0 (See Methods) and assumed that the values of P_AV_, P_AS_, P_SA_, P_SV_, P_VA_, and P_VS_ are randomly assigned from 0 to 1 so that a randomly chosen site can be transitioned into a site of a tSC, a vacancy, or an axon adjacent to the chosen site. The competition process was iterated by repeating the random selection of a transition site until there is no change in the composition of the sites. The simulation beginning with these initial layouts using random transition probabilities showed that the competition can lead to an endplate entirely occupied by only one type of axon after losing all other types of sites at the endplate sharply; however, the competition did not show the stable copresence of tSCs, vacancies, and a single axon after synapse elimination (Fig. 4). Moreover, other simulations led to an endplate filled only with tSCs or a completely unoccupied endplate (S1). Even with nonrandom, equal transition probabilities, synapse elimination did not reliably produce an endplate filled with one axon, tSCs, and vacancies where the values of P_AV_, P_AS_, P_SA_, P_SV_, P_VA_, and P_VS_ were all one-thirds (S2 and S3). Although some degrees of randomness and/or equal degrees in the interactions might be present during synapse elimination, our simulation results demonstrated that such randomness or equal fitness is insufficient to reliably recapitulate synapse elimination. Accordingly, we assumed that tSCs, vacancies, and axons have different fixed transition probabilities at least during a portion of this period of time. To determine their constant transition probabilities, we developed a stochastic model of synapse elimination that employs the areas of tSCs, vacancies, and axons at different postnatal days and Markov Chain methods that are widely used in biological modeling studies^29,31–35^. Our model assumes that tSCs and axons compete against each other as proposed from a previous study^7^, but here we newly propose that the competition of tSCs and axons is mediated by vacancies, and different types of axons compete to take over their adjacent vacancy in an endplate (Fig. 5). Specifically, the model assumes that vacancies mediate the transition between axonal and tSC sites without their direct transition (Fig. 5), and different axon types compete to occupy nearby sites. These assumptions allow us to describe the stochastic competition process as a Markov process^29,34^. Accordingly, we have the following equations:

1 $${{\rm{P}}}_{{\rm{AA}}}+{{\rm{P}}}_{{\rm{AV}}}=1$$

2 $${{\rm{P}}}_{{\rm{SS}}}+{{\rm{P}}}_{{\rm{SV}}}=1$$

3 $${{\rm{P}}}_{{\rm{VA}}}+{{\rm{P}}}_{{\rm{VS}}}=1$$

**Figure 6:**
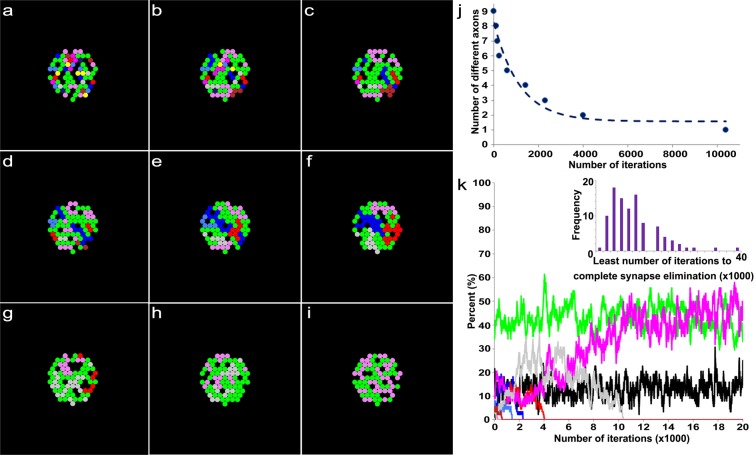
An example result based on a model of synaptic competition among axons, tSCs, and vacancies with different constant transition probabilities derived from the area ratios at P3 of the NMJs. Simulations carried out using the same configuration as described in Fig. 3, but the final ratios of tSCs, vacancies and axons are set to be 0.57, 0.18, and 0.25, respectively, which are the ratios at P3 of the NMJs. (**a**) Initially, 9 different axons, tSCs and vacancies form their contact sites randomly on a muscle fiber with the initial ratio of their total contact areas (axons:tSCs:vacancies), which is about 30:16:54 as Fig. 4. (**b–h**) The competition among axons, tSCs, and vacancies with their constant transition probabilities shows elimination of multiple contact sites formed by different axons, tSCs, and vacancies at different iterations of the simulation (100, 200, 300, 700, 1500, 3400, and 4100 iterations, respectively). (**i**) When the simulation is at 10,400 iterations, the competition leads to a complete synapse elimination with tSC and vacant sites present demonstrating that optimal transition probabilities reliably simulate synapse elimination. (**j**) The number of different types of axons in the endplate reduces sharply as the iteration proceeds. (**k**) Change in the ratios of the contact areas formed by tSCs (green), vacancies (black), and 9 different axons (colors different from green and black) as the simulation based on the stochastic model of tSC and vacancy mediated synapse elimination proceeds. Inset: the distribution of the least number of iterations when synapse elimination is complete from 100 repeated simulations.

**Figure 7:**
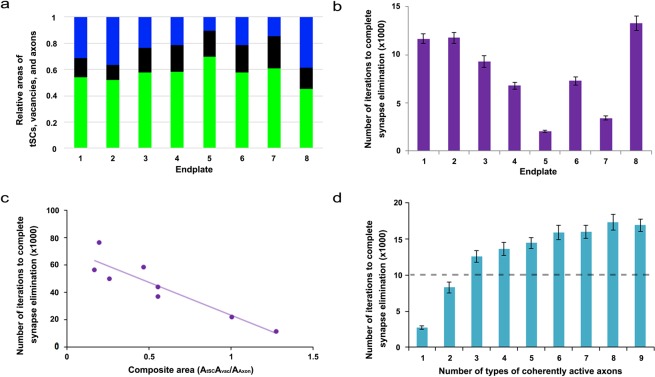
Relationships of the simulated average least number of iterations to complete synapse elimination with different ratios of contact areas of tSCs, vacancies, and axons and with synaptic activity. (**a**) Measured average ratios of contact areas of tSCs (green), vacancies (black), and axons (blue) from each of the 8 different endplates at P3. (**b**) The average least number of iterations to complete synapse elimination for each of the measured ratios by repeating the simulations 100 times for each. Error bars are standard errors. (**c**) Negative correlation of the composite area of (**a**) with the least number of iteration to complete synapse elimination (Spearman correlation, p < 0.05). (**d**) Synaptic activity dependent synapse elimination. The simulation repeated 100 times as the number of active axons increased from 1 to 9. The average number of iterations to complete synapse elimination ratio obtained with the average ratio of the areas at P3 (57:18:25) was used as a reference rate of synapse elimination for comparison, and it is represented as a dotted line. When one to two different axons out of nine different axons in the endplate were active, synapse elimination accelerated. In contrast, when more than two different nerves were active, synapse elimination slowed down. Error bars are standard errors.

There are only 3 independent transition probabilities (P_AV_, P_SV_, and P_VS_) because P_AA_, P_SS_, and P_VA_ depend on P_AV_, P_SV_, and P_VS_ based on the assumed relationship in Eqs. 1–3. In addition, it is assumed that each synaptic site can be occupied by a neighboring axon consistent with other and our studies^7,8,26^. Thus, when an individual axon occupies multiple neighboring sites near a recently vacated site, the axon has a higher probability of reclaiming that vacancy than other axons with fewer sites proximal to the vacated site. As in the previous section, we constructed the initial layout of the endplate composed of multiple contact sites made by tSCs (green), vacancies (black), and axons (other colors) using the empirical data at P0. Again these initial layouts (Fig. 6a,f,k) depict the relative ratio of the contact sites’ areas measured in the P0 data sets and randomly distribute tSC, vacancy, and axon sites in an endplate. Then, the competition process among them was iterated by repeating the random selection of a transition site in the layout until only one axon (identified by color) remained. As shown in Fig. 6, the model with fixed transition probabilities, determined using the measured relative areas of axons, tSCs, and vacancies at P3, reliably reproduced synapse elimination. The results showed that the number of different axons decreases exponentially consistent with other studies^3,8,15^. The results also showed that the contact sites of tSCs, vacancies, and axons in an endplate interchange dynamically as different axons compete against each other (Fig. 6); when synapse elimination is complete, the endplate is occupied by tSCs, vacancies, and only one axon (Fig. 6i–k). The rate of synapse elimination is known to vary between different muscles and even between fibers in the same muscle^14^. Consistently, when we repeated the simulation 100 times, the number of iterations varied broadly (the inset of Fig. 6k); the average iteration to complete synapse elimination was 10300 ± 6719 (SD). Furthermore, direct measurements of the relative ratios of tSCs, vacancies, and axons at P3 were different from one endplate to another (Fig. 7a). Accordingly, it is reasonable to expect that our model using the different ratios may exhibit variable rates of synapse elimination. As expected, simulation results of our model with the different ratios of the 8 measured endplates showed that synapse elimination is complete at very different numbers of iterations, thus variable rates of synapse elimination (Fig. 7b). Despite such broad variation, we noted that the least number of iterations to complete synapse elimination is less on average with large relative areas of tSCs and/or vacancies than those with small relative areas (Fig. 7b) suggesting that tSC and vacancy occupation are closely related to the rate of synapse elimination.

### Synapse elimination correlated with relative areas of tSCs, vacancies, and axons

To examine the correlation of the rate of synapse elimination with the relative areas of tSCs, vacancies, and axons with regard to their interdependence, we combined the relative areas into a composite area by dividing the product of the area of tSCs and that of vacancies by that of axons. The composite area was found to be negatively correlated with the least number of iterations to complete synapse elimination (Spearman Correlation, p < 0.05) as shown in Fig. 7c; the same correlation analysis using manually adjusted relative areas also showed that the composite area is negatively correlated with the least number of iterations to complete synapse elimination (Spearman Correlation, p < 0.05) as shown in S4 indicating that the composite area is positively correlated with the rate of synapse elimination. It is also reasonable to expect that the rate of synapse elimination decreases over time because the synapse elimination is complete in several weeks after birth. As expected, the simulation using the average ratio at P7 showed that synapse elimination is complete after 15700 iterations on average, which is markedly greater than that at P3 (S5). The average least numbers of iterations to complete synapse elimination between P3 and P7 were found to be significantly different (n = 100, t-test p < 0.05) indicating that the rate of synapse elimination is slower at P7 than P3. Similarly, simulations using average ratios at P16 showed completed synapse elimination after 15000 iterations on average, also greater than that at P3 (S6). The average least number of iterations to complete synapse elimination were significantly different between P3 and P16 (n = 100, t-test p < 0.05) indicating that the rate is less at P16 than that at P3. Our results are consistent with our expectation of a decreasing rate of synapse elimination over time. The average iterations of the complete synapse elimination between P7 and P16 were not significantly different (t-test, p = 0.59), which may suggest that the rate has already reached its minimum around P7.

### Synaptic activity dependent selective and accelerated synapse elimination

It is widely held that synaptic activity is closely related to normal synapse elimination; several experimental studies support that axons having higher synaptic activity tend to remain at their endplate^37–40^. To test whether our model can predict this activity dependent synapse elimination, we assumed that among 9 different axons, one, selected at random, is more active than the others and the active axon sites have two-fold lower probability of having a site that undergoes the transition than other sites (See Methods). We generated the initial layouts using the average relative ratios of tSCs, vacancies, and axons at P0 and determined their transition probabilities using the average relative areas at P3 in the same way with the previous sections. Our simulation results showed that the active axon was the winner with the probability of 97%, consistent with the studies on synapse elimination and synaptic activity. Furthermore, the results showed that synaptic activity accelerates the rate of synapse elimination four-fold compared to that of control NMJs (Fig. 7d). When two axons were set to be more active than the others, our simulations predicted that the winning axon came from the active axons with the probability of 98%. Additionally, two active axons further accelerated synapse elimination but only by 10%, indicating that multiple coherently active axons in an endplate weakly promotes competition leading to little or no additional acceleration of synapse elimination (Fig. 7d). Increasing the number of active axons slowed synapse elimination and the rate became even less than that when no axons were more active (Fig. 7d). This demonstrates that our model can account for synaptic activity dependent axon selection, and the acceleration and delay of synapse elimination it predicts are consistent with other experimental studies^37,39,41–43^.

## Discussion

This study developed a stochastic model of synapse elimination that, for the first time, incorporates classical interaxonal competition with the recently proposed competition between tSCs and axons, predicting that the competition is mediated by vacancies, a third endplate occupant. With serial electron micrographs from 25 different developing mouse NMJs, the model employed the relative contact areas of tSCs, vacancies, and axons on muscle fibers as key parameters to determine the degrees of their interaction. Simulation results of our model reliably exhibited coexistence of axons with tSCs and vacancies during synapse elimination and even after synapse elimination was complete. Furthermore, our model successfully simulated expedited synapse elimination with synaptic activity, and predicts that the rate of synapse elimination varies during development suggesting that the ratios of the contact areas of these structural components are important to understand the roles of the components in synapse elimination, the mechanisms underlying impaired and enhanced synapse elimination, and the development of neural systems.

### Interaxonal competition and competition between axons and tSCs for their adjacent vacancies

A competitive process among axons innervating the same muscle fiber is thought to play an important role in synapse elimination. At P0 each axon occupies a similar proportion of the endplate area, so it is impossible to determine which axon will remain after synapse elimination by comparing their areas at this stage. Within days, however, some axon sites are vacated and the vacant sites taken over by different axons leading to the eventual loss of all but one axon. Accordingly, it is reasonable to assume that a vacancy can form randomly by the removal of an axon from the endplate of a muscle fiber. Any axons in proximity are expected to compete for the newly vacated site. Such competitive processes have been observed in experimental studies and examined theoretically in several modeling studies^8,15,22^. Specifically, in a model based on evolutionary graph theory, synaptic rearrangement occurs in a neuromuscular junction filled with several different axons; after random removal of sites, neighboring axons with equal fitness compete for the vacated sites, the model successfully simulated the competitive process of synapse elimination^8^. However, a role for Schwann cells, which occupy the endplate alongside axons, in synapse elimination^7,26^ is not considered in the model. Since the model is designed to allow only the winning axon to remain after synapse elimination, the coexistence of tSCs and vacancies with it is neither accounted for nor possible (See Figs. 5, S1, S2, and S3). While previous studies provided indirect evidence for Schwann cell-axon competition during synapse elimination and propose a qualitative model of Schwann cell mediated synapse elimination^7^, this and other studies did not provide a quantitative model to characterize such a direct role for Schwann cells. With respect to vacancies as mediators of competition, we assumed random occurrence of a vacancy in a muscle fiber endplate similar to random competition models of different axons for synapse elimination^8^. Furthermore, we extend the random competition to tSCs by assuming that a vacancy can form by removing an axon or a tSC from an endplate. It is reasonable to assume that a vacancy can be formed by removing a tSC from an endplate with the concomitant competition for the vacated site if a random competitive process occurs between tSCs and axons^7,26^. We assumed that their transition probabilities are closely related to their relative contact areas as determined using their relative areas based on Markov Chain methods, increasingly used to understand the probabilistic behavior of the dynamic stochastic mechanisms associated with biological processes^34^. According to our model, axons and Schwann cells do not have equal fitness for taking over vacancies, and assignment of equal or random fitness rendered synapse elimination unreliable (See S1, S2, and S3). A recent study reported that the relative area occupied by tSCs at P3 is greater than the area at P0 and also that at P16^7^, proposing a mechanism of synapse elimination in which tSCs directly compete against axons to take over an endplate. A study by others examining NMJs of soleus muscles of P7-P8 mouse reported that tSCs have different Ca^2+^ responses to weak and strong axons, and proposed another mechanism whereby tSCs differentiate weak axons from strong axons and are involved in selectively removing weak ones, supporting the active role of tSCs in synapse elimination^44^. Furthermore, a recent study reported that overexpression of NRG1-III enhanced tSC activity and expedited synapse elimination while reduced NRG1-III slowed synapse elimination^26^. Those studies raise the possibility that increases in the relative area of tSCs may speed up synapse elimination. When we modified the relative area of tSCs in our models, simulations reflected an enhanced rate of synapse elimination, consistent with the study. Furthermore, our model predicted that an increase in the relative area of vacancies would enhance synapse elimination more significantly than increasing tSC area alone. Based on the results, it is expected that relative areas of vacancies are large during the most active phase of synapse elimination. As expected, our measurement of the areas showed that the large relative area of vacancies maintained until P3 whereas the large relative area of tSCs appears between P3 and P7. Such differences may be closely related to the level of tSC activity and tSC-involved mechanisms of regulating the rate of synaptic elimination. As the relative areas of tSCs and vacancies change during synapse elimination, our model suggests that the rate of synapse elimination also varies accompanying the change.

### Synaptic activity dependent synapse elimination

Synaptic activity is known to affect the rate of synapse elimination, and a number of studies have concluded that although synaptic activity itself is not a sole decisive factor of synapse elimination^45–47^, relatively active axons have competitive advantage^37,41,48–61^. Presynaptic block by tetrodotoxin and botulinum toxin and postsynaptic block by α-bungarotoxin and curare led to the prolonging of synapse elimination^52–56^. Electrical stimulation of the nerves of 6–7 day old rat pups at 8 Hz for 4–6 hours per day for 2–4 days was found to decrease the level of polyinnervation from ~85% to ~50%^37^; furthermore, electrical stimulation of muscles also was found to accelerate synapse elimination^39^. From these studies, it is reasonable to expect that enhancing synaptic activity accelerates synapse elimination and provides the active axon with an advantage. Consistent with prior studies, our simulation results showed that an active axon is more likely to win sole occupancy of the endplate and that such synaptic activity accelerates synapse elimination. Furthermore, our model predicted that when two different axons have the same degree of high synaptic activity compared to other axons, the effect of accelerating synapse elimination is significantly weakened. Our model also predicted that when more than two different axon types have the same degree of high synaptic activity, synapse elimination slows rather than accelerates, suggesting that multiple coherently active axons delay synapse elimination instead of accelerating it. Although our simulated high synaptic activity of an axon or more is difficult to be converted into a specific stimulation frequency currently, our results are consistent with several other studies reporting that asynchronous synaptic activity promotes synapse elimination whereas synchronous synaptic activity delays it^41,46,57,58,60,61^.

**Figure 8:**
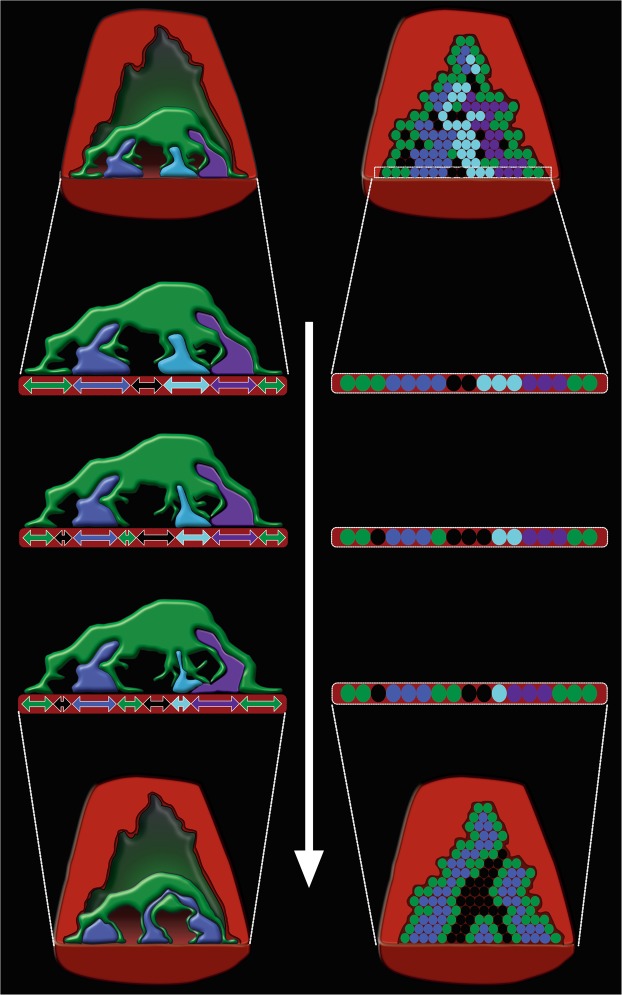
A stochastic model of tSC and vacancy mediated synapse elimination. According to the model, a vacant site or vacancy in an endplate of a muscle fiber forms randomly by a removing tSC or nerve terminal of an axon. The transition probability of a tSC to a vacancy and an axon to a vacancy are determined by measured relative areas of tSCs, vacancies, and axons. Subsequently, the vacancy is taken over by adjacent axons or tSCs. But the probability of its taking over by a specific axon is positively correlated with the axon’s relative contact area surrounding the vacancy. In other words, the greater the contact area of an axon surrounding the vacancy is, the greater the probability of its taking over the vacancy is. When the synaptic activity of an axon is greater than other axons in an endplate, the probability of the axon’s taking over is greater than other axons leading to acceleration of synapse elimination consistent with previous and other studies. Furthermore, our model newly predicts that as the relative area of the vacancy or tSC in an endplate increases synapse elimination speeds up raising a testable hypothesis that both the measurable relative areas of vacancies and tSCs are important structural correlates of the rate of synapse elimination.

As with any model of a biological process, it is impossible to describe all factors in play. For example, intrinsic differences in fitness among multiple axons may be present. Kasthuri and Lichtman proposed that motor neurons innervating fewer muscle fibers than others are more competitive than others^62^. Since our model predicts that the axons of the more competitive neurons tend to possess greater contact areas than others, measurement of the areas at a specific postnatal day such as P0 can be used to test the proposed idea by examining the distribution of the areas. The contribution of muscle fibers, such as neurotrophic factor release or removal of acetylcholine receptors^38,39^, may influence the process of synapse elimination. Increase or decrease of histocompatibility complex class 1 molecules and N-Methyl-D-aspartic acid (NMDA) receptors has been shown to accelerate or delay synapse elimination, respectively^63,64^. In the case the increase in their level is expected to enlarge the areas of vacancies and/or tSCs following the accelerated synapse elimination whereas the decrease in the level is expected to reduce those areas following delayed synapse elimination. But the intrinsic differences, contribution of muscle fibers, and other factors known to be implicated in synapse elimination are not considered in our model because there are no reliable structural correlates with them yet. Despite these limitations, our stochastic model (See Fig. 8) reliably simulates synapse elimination, agrees well with the observed coexistence of Schwann cells, vacancies and axons at endplates at its conclusion, and provides an explanation of the different dependences of synapse elimination on synchronous and asynchronous synaptic activities. Furthermore, the model reveals a novel role for vacant sites in synapse development closely related to the competition between tSCs and axons. Testing the predictions made by the stochastic model of synapse elimination at single and multiple endplates would contribute to our more complete understanding of synapse elimination and offer insights into the nature of synapse development and network refinement.

## Methods

### Tissue preparation and imaging for serial-section transmission electron microscopy

20 different serial electron microscopy data sets from sternomastoid muscles of wild-type C57B/6 mice of either sex obtained from a previous study^7^ were analyzed. The mice were obtained from the Jackson Laboratory, bred in a colony at the University of Texas at Austin and Texas A&M at College Station, and killed on the day of birth (P0; n = 10; 5 mice), the third postnatal (P3; n = 8; 3 mice), and the sixteenth postal natal days (P16; n = 2; 2 mice). Experimental procedures were approved by the Institutional Animal Care and Use Committees at the University of Texas and Texas A&M University and all experiments and methods were performed in accordance with the relevant guidelines and regulations. The generation of wild-type mice and their sample preparation for electron microscopy for mice of P0, P3, and P16 have been described previously^7,65,66^. Simply, animals were perfused transcardially with 0.1 M sodium cacodylate buffer, pH 7.4, followed by the same buffer containing 2% paraformaldehyde and 3% glutaraldehyde. Sternomastoid muscles were removed and fixed overnight at room temperature in the same fixative. Muscles were washed with cacodylate buffer and stained en bloc in 1% osmium tetroxide, 1% ferrocyanide in cacodylate buffer for 5 h, washed with water, and then stained in 1% aqueous uranyl acetate for 2 h. Muscles were dehydrated in graded alcohols and acetone, then embedded in Epon 812. Regions containing NMJs were located by thick (0.5–1 µm) sections made on a Leica Ultracut UTC Ultramicrotome with glass knives, stained with 1% toluidine blue, and examined under a light microscope. Blocks were further trimmed using a 45 degree cryotrim tool (Diatome) and serial 65 nm (silver) sections cut with a 35 degree diamond knife (Diatome). Sections were mounted on formvar-coated Synaptek slot grids (Electron Microscopy Sciences) and observed under a FEI Tecnai Spirit electron microscope with an AMT Advantage HR digital camera. NMJs were identified by the apposition of nerve terminals to within ~50 nm of the muscle fiber surface in the presence of Schwann cells. Digital images of individual NMJs and the immediately surrounding area were captured from each section at a magnification of 16,500×. At this magnification, ~6–12 overlapping images were acquired to include the entire extent of the NMJ. These individual images were then montaged by manual manipulation in Adobe Photoshop to create a single image. Such montaged images over 70–150 serial sections were then imported into the software program Reconstruct^67^ and calibrated based on section thickness and known distances in the specimen. In software, the montage in each section was aligned linearly to each adjacent montage using 6–8 objects common in both and starting with the central montage in the stack and working toward each the first and last montage. Once aligned, structures of interest in each section were segmented (traced) using the tools provided in the software. Measurements and 3D renderings of these structures were then generated using Reconstruct.

### Tissue preparation and imaging for serial block-face scanning electron microscopy

Tissues of a mouse at the seventh postnatal day (P7) were prepared differently and imaged using serial-block-face scanning electron microscopy (SBSEM). Tissue preparation and staining was done with compliance to Renovo Neural Inc. (Cleveland, OH). Briefly, P7 C57/Bl6 mice were transcardially perfused with 2.5% glutaraldehyde and 4% paraformaldehyde in a 0.1 M sodium cacodylate buffer. Sternomastoid muscles were then removed and stored in perfusion solution for 1 day prior to staining. Tissue was then stained with uranyl acetate and osmium-ferrocyanide, followed by graded-dehydration and embedded in Epon resin. SBSEM image data sets of postnatal day 7 NMJs were acquired at Renovo Neural Inc. (Cleveland, OH). A sample image is shown in Fig. 1e. The tissue blocks were mounted, examined, and sectioned in a Zeiss Sigma VP scanning electron micro-scope equipped with a Gatan 3View in-chamber ultra-microtome stage with low-kV backscattered electron detectors optimized for 3View systems. NMJs were initially identified within cross-sections of muscle tissue by axon terminals in direct apposition to junctional folds on the muscle fiber membrane and capped by terminal Schwann cells. Regions of interest were chosen to include the NMJs in whole cross-section. The first sample block was sectioned cross-sectionally along the long axis of the muscle fibers. A series of 465 EM images in a field size of 130 µm × 50 µm were acquired at 2.2 kV with a resolution of 8.1 nm per pixel and 90 nm per slice. Eight NMJs were captured at P7 with 5 of those NMJs fully reconstructed for synaptic contact areas.

### A model of tSC and vacancy mediated synapse elimination and its simulation

We used circular contact sites assuming that all of the sites are composed of 9 different axons, which were the maximum number of different axons observed at P0, tSCs, and vacancies consistent with our observation of them from 2D scanning electron microscope images, and their initial layout is generated based on the recently reported ratios of contact areas of the tSCs, vacancies, and axons at P0. To simulate the initial layout, it is assumed that the total area is covered by tSCs about 30%, vacant sites about 16%, and axons about 54%, based on their recently measured ratios at P0. All of the sites are randomly formed within a round region of a diameter of 300 pixels, and each site has a diameter of 30 pixels as shown in Figs. 4 and 6 and all the supplemental figures. Vacant sites are black, tSC sites green, and axonal sites in different colors (blue, red, dark blue, yellow, pink, purple, dark green, gray, and brown). Thus, each of the 9 different axons takes up about 6% of the total area. Examples of randomly generated initial layouts at P0 are shown in Figs. 4a and 6a. We used the optimal probabilities accounting for the synapse elimination and all the ratios of different kinds of synaptic sites using Markov chain properties^30^ assuming that the ratio of their contact areas with a muscle fiber in a NMJ reflects the efficiency of synapse elimination. We noted that at P3, the ratio of the contact areas of tSC sites greatly increased and gradually decreased later on raising a possibility that the ratio of the areas of tSC sites plays an important role on the process of synapse elimination consistent with the recent study^7^. Accordingly, the measured ratio of the contact areas of tSC sites, vacancies, and axonal sites at P3 was used here to obtain the optimal transition probabilities by determining the probabilities that can stabilize the ratio using Eq. 4 based on the Markov Chain properties^30^.

4 $${\overrightarrow{r}}_{f}\,[\begin{array}{ccc}{P}_{SS} & {P}_{SV} & 0\\ {P}_{VS} & 0 & {P}_{VA}\\ 0 & {P}_{AV} & {P}_{AA}\end{array}]={\overrightarrow{r}}_{f}\,[\begin{array}{ccc}{P}_{SS} & 1-{P}_{SS} & 0\\ {P}_{VS} & 0 & 1-{P}_{VS}\\ 0 & {P}_{AV} & 1-{P}_{AV}\end{array}]={\overrightarrow{r}}_{f}$$

where ${\overrightarrow{r}}_{f}$, a vector having the final ratios. We have used the average ratios of the developing NMJs at different postnatal days ranging from P0 to P16 as shown in Table 1.

**Table 1:** Average ratios of the contact area of tSCs, mouse NMJs measured at P0, P3, P7, and P16 (n = 10, 8, 5, and 2, respectively).

Postnatal days	tSC	Vacancy	Axon
P0	31%	17%	52%
P3	57%	18%	25%
P7	55%	5%	40%
P16	41%	6%	53%

Then, transition probabilities were adjusted to generate the final ratios close to those obtained at P3 from our previous study (${\overrightarrow{r}}_{f}$^7^;) using multiple NMJs of neonatal mice. According to Eq. 4, two independent variables of all the three independent variables can be expressed in terms of the remaining independent variable. Thus, there is only one undetermined transition probability in the matrix. We chose the transition probability from a vacancy to a tSC site (*P*_*VS*_) as the undetermined probability. As *P*_*VS*_ is varied, the rest of the transition probabilities are determined using the value of *P*_*VS*_ and the ratios at P3. Then, for each specific *P*_*VS*_, the process of the synaptic elimination was iterated until it was complete. But it was noted that for some values of *P*_*VS*_ a few other transition probabilities happened to be negative or undetermined. Thus, the value of *P*_*VS*_ was set as 0.6, which allowed us to use all of our measured ratios of tSCs, vacancies, and axons such as those of multiple mouse NMJs at P3, P7, and P16 and ensured that all the other transition probabilities were reliably computed from our measured ratios; the range of the probabilities was broad from ~0.4 to ~0.7. For the specific *P*_*VS*_, the process of the synaptic elimination was iterated until it was complete or the number of iterations reached a specified maximum number of iterations that was sufficient for completing the synapse elimination (See Figs. 6–7, S5 and S6).

To simulate the synaptic activity dependent synapse elimination, the initial layout was constructed in the same way. By assuming that only a type of axons is active in an endplate, all the sites of the active axon among 9 different axons were selected and we set the probability of each of the sites to have a chance to undergo the transition to be two times less than each of all sites excluding the active axon; then as described above, the process of synapse elimination was iterated until it was complete or the number of iteration reached a specified maximum number of iteration that is sufficient for completing the synapse elimination (See Fig. 7). We also assumed that more than one type of axons are active in an endplate. In the case, we set the probability of having the transition site in each site of those active axons to be two times less than each of all sites excluding the active axons similar to the case of a single active axon (See Fig. 7d).

### Simulations based on random transition probabilities

Because each transition probability might fluctuate randomly, first all of the probabilities are assumed to be random ranging from 0 to 1. In each step of the simulation, a contact site from all the sites in an endplate was randomly chosen. Then the site is converted into a different site with randomly assigned transition probabilities while the relationships of the probabilities as described in Fig. 3b and its legend are maintained. The step was repeated more than 10000 times to ensure that only one type of axonal site survived at most as shown in Figs. 4 and S1.

### Simulations based on equal transition probabilities

We assumed that all of the probabilities are fixed and the same. Using all the sites randomly assigned in the same way described above, at each step a contact site was randomly chosen from them and transitioned using the transition probabilities of neighboring axons, tSCs or vacancies where all of the probabilities are equal to one third. The process was repeated 200000 to make sure that only one type of synaptic site survived as shown in S2 and S3.

### Computer hardware and software

In order to perform the simulations, a PC computer was used loaded with Windows 7, IDL (Harris Geospatial Solutions, Broomfield, CO, USA) and a program written in IDL based on the Markov chain model described previously was employed for the simulations.

### Statistical analyses

Two-tailed Student’s t-tests and Spearman rank correlation tests were performed with OriginPro (OriginLab, Northampton, MA, USA). Significance was defined as the p value, p, which is less than 0.05. All the averages were given with their standard deviation (SD) unless noted.

## Supplementary information

Supplementary Information

## Data Availability

All data supporting the findings of this study are available from the authors upon reasonable request.

## Code availability

Code from this study (IDL scripts) is available from the corresponding author upon request.

## References

[1] Hall ZW, Sanes JR (1993). Synaptic structure and development: the neuromuscular junction. Cell.

[2] Sanes JR, Lichtman JW (1999). Development of the vertebrate neuromuscular junction. Annu Rev Neurosci.

[3] Brown MC, Jansen JK, Van Essen D (1976). Polyneuronal innervation of skeletal muscle in new-born rats and its elimination during maturation. J Physiol.

[4] Darabid H, Perez-Gonzalez AP, Robitaille R (2014). Neuromuscular synaptogenesis: coordinating partners with multiple functions. Nat Rev Neurosci.

[5] Purves D, Lichtman JW (1980). Elimination of synapses in the developing nervous system. Science.

[6] Redfern PA (1970). Neuromuscular transmission in new-born rats. J Physiol.

[7] Smith IW, Mikesh M, Lee Y, Thompson WJ (2013). Terminal Schwann cells participate in the competition underlying neuromuscular synapse elimination. J Neurosci.

[8] Turney SG, Lichtman JW (2012). Reversing the outcome of synapse elimination at developing neuromuscular junctions *in vivo*: evidence for synaptic competition and its mechanism. PLoS Biol.

[9] Van Essen DC, Gordon H, Soha JM, Fraser SE (1990). Synaptic dynamics at the neuromuscular junction: Mechanisms and models. Journal of Neurobiology.

[10] van Ooyen A, Willshaw DJ (1999). Poly- and mononeuronal innervation in a model for the development of neuromuscular connections. J Theor Biol.

[11] Walsh MK, Lichtman JW (2003). *In vivo* time-lapse imaging of synaptic takeover associated with naturally occurring synapse elimination. Neuron.

[12] Wu H, Xiong WC, Mei L (2010). To build a synapse: signaling pathways in neuromuscular junction assembly. Development.

[13] Betz WJ, Caldwell JH, Ribchester RR (1979). The size of motor units during post-natal development of rat lumbrical muscle. J Physiol.

[14] Jansen JK, Fladby T (1990). The perinatal reorganization of the innervation of skeletal muscle in mammals. Prog Neurobiol.

[15] Barber MJ, Lichtman JW (1999). Activity-driven synapse elimination leads paradoxically to domination by inactive neurons. J Neurosci.

[16] Gouze JL, Lasry JM, Changeux JP (1983). Selective stabilization of muscle innervation during development: a mathematical model. Biol Cybern.

[17] Bennett MR, Robinson J (1989). Growth and elimination of nerve terminals at synaptic sites during polyneuronal innervation of muscle cells: a trophic hypothesis. Proc R Soc Lond B Biol Sci.

[18] Rasmussen CE, Willshaw DJ (1993). Presynaptic and postsynaptic competition in models for the development of neuromuscular connections. Biol Cybern.

[19] Jeanpretre N, Clarke PG, Gabriel JP (1996). Competitive exclusion between axons dependent on a single trophic substance: a mathematical analysis. Math Biosci.

[20] Elliott T, Shadbolt NR (1996). A mathematical model of activity–dependent, anatomical segregation induced by competition for neurotrophic support. Biological Cybernetics.

[21] Elliott T, Shadbolt NR (1998). Competition for Neurotrophic Factors: Mathematical Analysis. Neural Computation.

[22] Willshaw DJ (1981). The establishment and the subsequent elimination of polyneuronal innervation of developing muscle: theoretical considerations. Proc R Soc Lond B Biol Sci.

[23] Smalheiser NR, Crain SM (1984). The possible role of “sibling neurite bias” in the coordination of neurite extension, branching, and survival. J Neurobiol.

[24] Stollberg J (1995). Synapse elimination, the size principle, and Hebbian synapses. J Neurobiol.

[25] Bishop DL, Misgeld T, Walsh MK, Gan WB, Lichtman JW (2004). Axon branch removal at developing synapses by axosome shedding. Neuron.

[26] Lee YI (2016). Neuregulin1 displayed on motor axons regulates terminal Schwann cell-mediated synapse elimination at developing neuromuscular junctions. Proc Natl Acad Sci USA.

[27] Lee YI, Mikesh M, Smith I, Rimer M, Thompson W (2011). Muscles in a mouse model of spinal muscular atrophy show profound defects in neuromuscular development even in the absence of failure in neuromuscular transmission or loss of motor neurons. Dev Biol.

[28] Song JW (2008). Lysosomal activity associated with developmental axon pruning. J Neurosci.

[29] Jung JH, Doniach S (2017). A stochastic model of active zone material mediated synaptic vesicle docking and priming at resting active zones. Sci Rep.

[30] Meyn, S. & Tweedie, R. L. *Markov Chains and Stochastic Stability*. (Cambridge University Press, 2009).

[31] Allen, L. J. S. *An introduction to stochastic processes with applications to biology*. (Pearson/Prentice Hall, 2003).

[32] Armond JW (2014). A stochastic model dissects cell states in biological transition processes. Sci Rep.

[33] Soltani M, Vargas-Garcia CA, Antunes D, Singh A (2016). Intercellular Variability in Protein Levels from Stochastic Expression and Noisy Cell Cycle Processes. PLoS Comput Biol.

[34] Wilkinson DJ (2009). Stochastic modelling for quantitative description of heterogeneous biological systems. Nat Rev Genet.

[35] Yakovlev A, Yanev N (2006). Branching stochastic processes with immigration in analysis of renewing cell populations. Math Biosci.

[36] van Ooyen A (2001). Competition in the development of nerve connections: a review of models. Network.

[37] O’Brien RA, Ostberg AJ, Vrbova G (1978). Observations on the elimination of polyneuronal innervation in developing mammalian skeletal muscle. J Physiol.

[38] Nelson PG, Fields RD, Yu C, Liu Y (1993). Synapse elimination from the mouse neuromuscular junction *in vitro*: a non-Hebbian activity-dependent process. J Neurobiol.

[39] Thompson W (1983). Synapse elimination in neonatal rat muscle is sensitive to pattern of muscle use. Nature.

[40] Magchielse T, Meeter E (1986). The effect of neuronal activity on the competitive elimination of neuromuscular junctions in tissue culture. Brain Res.

[41] Favero M, Busetto G, Cangiano A (2012). Spike timing plays a key role in synapse elimination at the neuromuscular junction. Proc Natl Acad Sci USA.

[42] Busetto G, Buffelli M, Tognana E, Bellico F, Cangiano A (2000). Hebbian mechanisms revealed by electrical stimulation at developing rat neuromuscular junctions. J Neurosci.

[43] Favero M, Buffelli M, Cangiano A, Busetto G (2010). The timing of impulse activity shapes the process of synaptic competition at the neuromuscular junction. Neuroscience.

[44] Darabid H, Arbour D, Robitaille R (2013). Glial cells decipher synaptic competition at the mammalian neuromuscular junction. J Neurosci.

[45] Callaway EM, Soha JM, Van Essen DC (1987). Competition favouring inactive over active motor neurons during synapse elimination. Nature.

[46] Costanzo EM, Barry JA, Ribchester RR (2000). Competition at silent synapses in reinnervated skeletal muscle. Nat Neurosci.

[47] Barry JA, Ribchester RR (1995). Persistent polyneuronal innervation in partially denervated rat muscle after reinnervation and recovery from prolonged nerve conduction block. J Neurosci.

[48] Balice-Gordon RJ, Lichtman JW (1994). Long-term synapse loss induced by focal blockade of postsynaptic receptors. Nature.

[49] Ribchester RR, Taxt T (1983). Motor unit size and synaptic competition in rat lumbrical muscles reinnervated by active and inactive motor axons. J Physiol.

[50] Ridge RM, Betz WJ (1984). The effect of selective, chronic stimulation on motor unit size in developing rat muscle. J Neurosci.

[51] Buffelli M (2003). Genetic evidence that relative synaptic efficacy biases the outcome of synaptic competition. Nature.

[52] Duxson MJ (1982). The effect of postsynaptic block on development of the neuromuscular junction in postnatal rats. J Neurocytol.

[53] Thompson W, Kuffler DP, Jansen JK (1979). The effect of prolonged, reversible block of nerve impulses on the elimination of polyneuronal innervation of new-born rat skeletal muscle fibers. Neuroscience.

[54] Ding R, Jansen JK, Laing NG, Tonnesen H (1983). The innervation of skeletal muscles in chickens curarized during early development. J Neurocytol.

[55] Srihari T, Vrbova G (1978). The role of muscle activity in the differentiation of neuromuscular junctions in slow and fast chick muscles. J Neurocytol.

[56] Brown MC, Hopkins WG, Keynes RJ (1982). Short- and long-term effects of paralysis on the motor innervation of two different neonatal mouse muscles. J Physiol.

[57] Fields RD, Nelson PG (1992). Activity-dependent development of the vertebrate nervous system. Int Rev Neurobiol.

[58] Personius KE, Balice-Gordon RJ (2001). Loss of correlated motor neuron activity during synaptic competition at developing neuromuscular synapses. Neuron.

[59] Sanes JR, Lichtman JW (2001). Induction, assembly, maturation and maintenance of a postsynaptic apparatus. Nat Rev Neurosci.

[60] Personius KE, Chang Q, Mentis GZ, O’Donovan MJ, Balice-Gordon RJ (2007). Reduced gap junctional coupling leads to uncorrelated motor neuron firing and precocious neuromuscular synapse elimination. Proc Natl Acad Sci USA.

[61] Buffelli M, Busetto G, Cangiano L, Cangiano A (2002). Perinatal switch from synchronous to asynchronous activity of motoneurons: link with synapse elimination. Proc Natl Acad Sci USA.

[62] Kasthuri N, Lichtman JW (2003). The role of neuronal identity in synaptic competition. Nature.

[63] Personius KE, Slusher BS, Udin SB (2016). Neuromuscular NMDA Receptors Modulate Developmental Synapse Elimination. J Neurosci.

[64] Tetruashvily MM, McDonald MA, Frietze KK, Boulanger LM (2016). MHCI promotes developmental synapse elimination and aging-related synapse loss at the vertebrate neuromuscular junction. Brain, behavior, and immunity.

[65] Li Y, Lee Y, Thompson WJ (2011). Changes in aging mouse neuromuscular junctions are explained by degeneration and regeneration of muscle fiber segments at the synapse. J Neurosci.

[66] Michailov GV (2004). Axonal neuregulin-1 regulates myelin sheath thickness. Science.

[67] Fiala JC (2005). Reconstruct: a free editor for serial section microscopy. J Microsc.

