# Supplementary material for: Terminal Schwann cell and vacant site mediated synapse elimination at developing neuromuscular junctions
Source: Sci Rep. 2019 Dec 9;9:18594. doi: 10.1038/s41598-019-55017-w (PMC6901572; doi:10.1038/s41598-019-55017-w)
Supplement: Supplementary file 1 — Supplementary Information [file 41598_2019_55017_MOESM1_ESM.pdf]

Terminal Schwann cell and vacant site mediated synapse elimination at developing neuromuscular junctions

Jae Hoon Jung[1,2*], Ian Smith[1,3], and Michelle Mikesh[4]

[1]Department of Biology, Texas A&M University, College Station, TX 77843

[2]Laboratory of Neurobiology, National Institute of Neurological Diseases and Stroke, National Institutes of Health, Bethesda, MD 20892

[3]Institute for Neuroscience, Texas A&M University, College Station, TX 77843

[4]Section of Molecular Cell and Developmental Biology, School of Biological Sciences, Institute of Cell and Molecular Biology and Neuroscience, University of Texas at Austin, Austin, TX 78712

[*]Correspondence to eurekajung@gmail.com

Supplemental Results

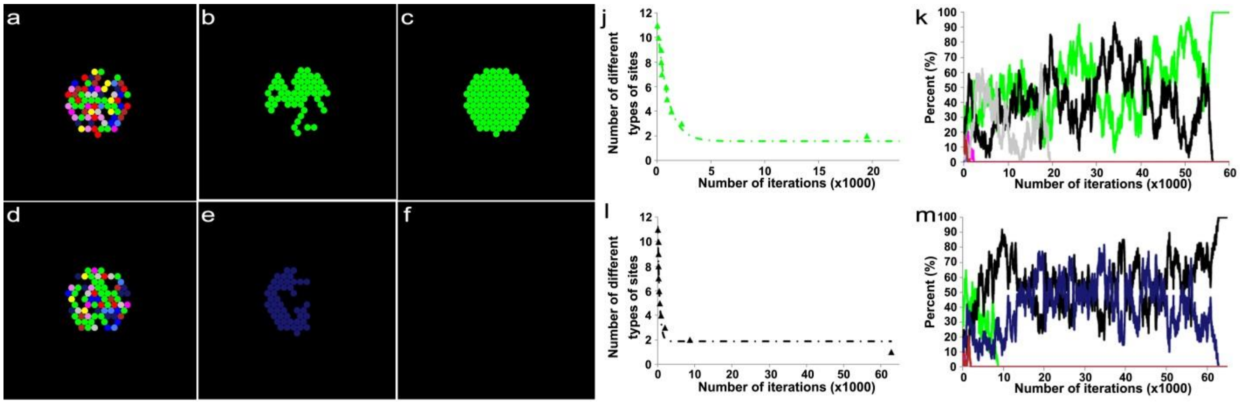

S1. Two different example results based on a model of synaptic competition among axons, tSCs, and vacancies with random transition probabilities. A green circle represents a synaptic site formed on a muscle fiber by a terminal Schwann cell (tSC). A black empty spot represents a vacant site or a vacancy having no axon or tSC on a muscle fiber. Circles having other 9 different colors represent synaptic sites formed on a muscle fiber by 9 different axons. (a, d) Initially, 9 different axons, tSCs and vacancies form their contact sites randomly on a muscle fiber. The initial ratio of their total contact areas (axons:tSCs:vacancies) is about 30:16:54, which was determined from a previous serial electron microscopy study on developing muscle fibers of mouse at P0 (See Methods). (b, e) The competition among axons, tSCs, and vacancies with their random transition probabilities shows elimination of multiple contact sites formed by different axons, tSCs, and vacancies when the iteration is 40000. (c, f) When the simulation is complete, the competition often leads to a complete synapse elimination. However, it can also generate contact sites formed by only tSCs or vacancies demonstrating that random transition probabilities among them cannot account for synapse elimination. (j, l) The number of remaining types of sites rapidly decreases consistent with other studies[1,2]. (k, m) The ratios of each type of contact sites formed by 9 different axons, a tSC, and a vacancy varies as the simulation proceeded with 60000 iterations showing that only one type of contact sites remains.

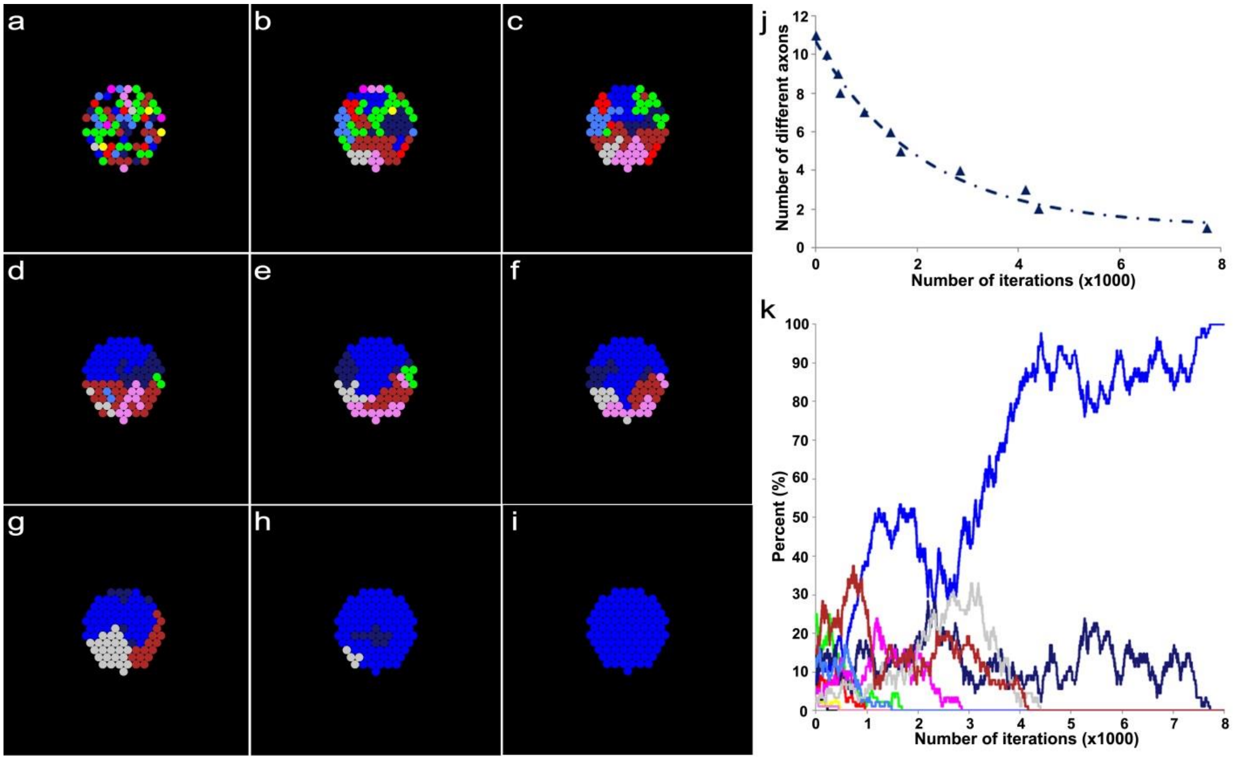

S2. An example result based on a model of synaptic competition among axons, tSCs, and vacancies with equal transition probabilities. (a) Initially, 9 different axons, tSCs and vacancies form their contact sites randomly in an endplate on a muscle fiber. The initial ratio of their total contact areas (axons:tSCs:vacancies) is about 30:16:54 as S1. (b-h) The competition among axons, tSCs, and vacancies with their random transition probabilities shows elimination of multiple contact sites formed by different axons, tSCs, and vacancies at different iterations of the simulation (300, 500, 1000, 1500, 1700, 2900, and 4200 iterations, respectively). (i) When the simulation reached 7800, synapse elimination is complete. However, only one type of axonal sites remains with no tSC and vacant sites that are present during and after synapse elimination of developing neuromuscular junctions. (j) The number of different types of axons in the endplate reduces as the iteration proceeds but decreases less sharply than those shown in S1 and Fig. 4. (k) Change in the ratio of the contact areas formed by tSCs (green), vacancies (black), and 9 different axons (colors different from green and black) as the simulation proceeds with 8000 iterations showing that only one type of contact sites remains.

.

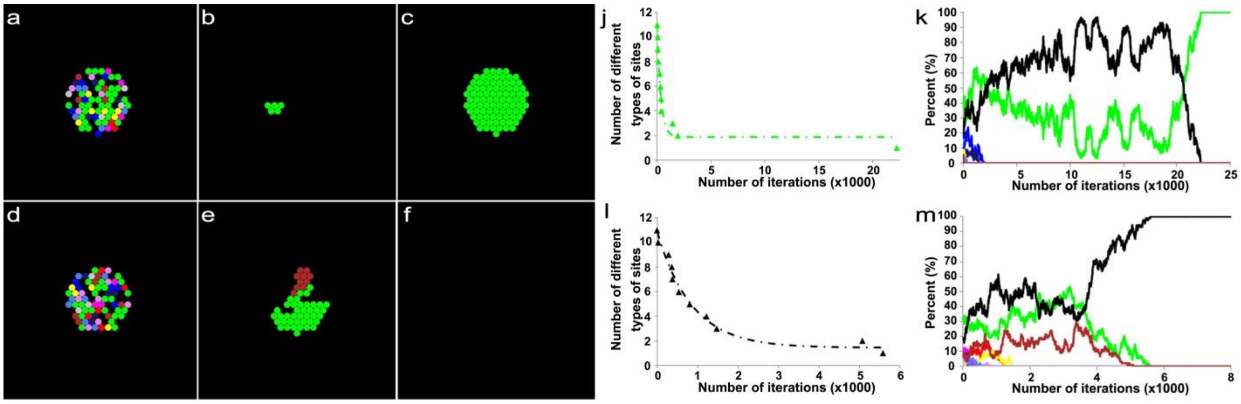

S3. Two different example results based on a model of synaptic competition among axons, tSCs, and vacancies with equal transition probabilities. A green circle represents a synaptic site formed on a muscle fiber by a terminal Schwann cell (tSC). A black empty spot represents a vacant site or a vacancy having no axon or tSC on a muscle fiber. Circles having other 9 different colors represent synaptic sites formed on a muscle fiber by 9 different axons. The simulations proceeded until synapse elimination was complete. (a, d) Initially, 9 different axons, tSCs and vacancies form their contact sites randomly on a muscle fiber. The initial ratio of their total contact areas (axons:tSCs:vacancies) is about 30:16:54 as S1 and S2. (b, e) The competition among axons, tSCs, and vacancies with their random transition probabilities shows elimination of multiple contact sites formed by different axons, tSCs, and vacancies when the iteration is 15000 and 4000, respectively. (c, f) When the simulation is complete, it often generates contact sites formed by only tSCs or vacancies demonstrating that random transition probabilities among them cannot account for synapse elimination. (j, l) The number of remaining types of axons rapidly decreases consistent with other studies. (k, m) The ratios of each type of contact sites formed by 9 different axons, a tSC, and a vacancy varies as the simulation proceeded with 25000 iterations and 8000 iterations, respectively, showing that only one type of contact sites remains.

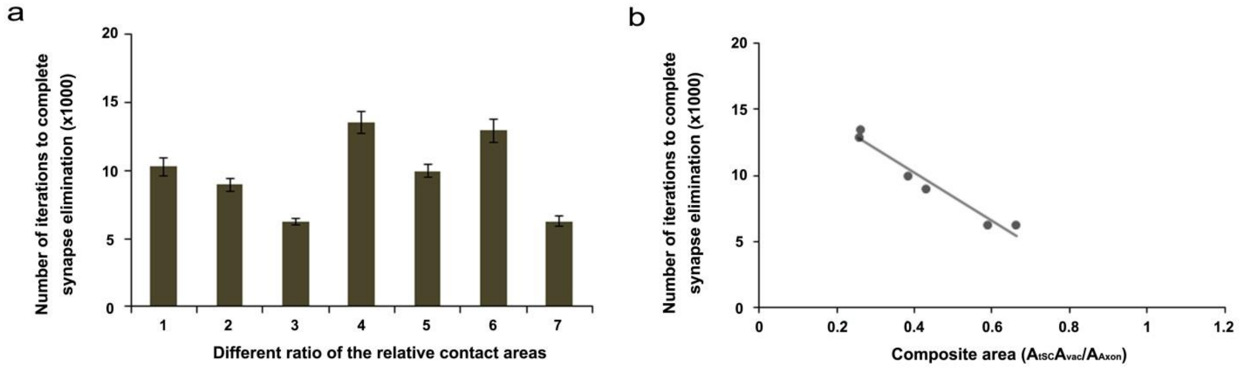

S4. Relationships of the simulated average least number of iterations to complete synapse elimination with arbitrarily adjusted ratios of contact areas of tSCs, vacancies, and axons. (a) The average least number of iterations to complete synapse elimination for each of the six arbitrarily different ratios of the contact areas by increasing or decreasing the portion of the area of tSCs, vacancies, and axons, respectively. The ratio at P3 (57:18:25) is used as a reference ratio for comparison (first bar). The changed ratios were 54:24:22 (second bar), 63:15:22 (third bar), 54:15:31 (fourth bar), 60:12:28 (fifth bar), 51:21:28 (sixth bar), and 60:21:19 (seventh bar). (b) Relationship of the composite area with the number of iterations to complete synapse elimination. All of the three fractions of the areas are combined into a variable called a composite area. The composite area is the product of the fraction of the area of tSCs and that of vacancies divided by that of axons. The composite area negatively correlates with the least number of iterations to complete synapse elimination (Spearman correlation, $p<0.05$).

Correlation of the rate of synapse elimination with relative areas of tSCs, vacancies, and axons.

These results in S4 show that an increase in the relative area of tSCs and/or vacancies accelerates synapse elimination in our model. We can then expect that an increase in the relative axon area will slow down synapse elimination. As expected, when we increased the relative ratio of the axons from 0.25 to 0.31 and decreased the ratio of tSCs and vacancies each by 0.03 (0.54 and 0.21 respectively), our simulation showed that the rate of synapse elimination decreased by about 28%, indicating that the ratio of the axons has a negative correlation with the rate of synapse elimination (S4a). We then decreased the relative ratio of the vacancies from 0.18 to 0.12 and increased the ratio of tSCs and axons each by 0.03 to keep their relative ratios constant (0.60 and 0.28 respectively). Our simulation showed that the rate of synapse elimination decreased by about 36% (S4a), which indicates again that the ratio of the vacancies has a positive correlation with the rate of synapse elimination. As we decreased the relative ratio of the tSCs from 0.57 to 0.51 and increased the ratio of the vacancies and axons each by 0.03 (0.21 and 0.28, respectively), we observed a decrease in the rate of synapse elimination of about 4%. This indicates that tSC ratio has a positive correlation with the rate of synapse elimination, but not as strong as that of vacancies, consistent with previous results (S4a). Finally, when we decreased

the relative ratio of the axons from 0.25 to 0.19 and increased the ratio of tSCs and vacancies each by 0.03 (0.54 and 0.21 respectively), our simulation showed that the rate of synapse elimination increased by about 57%, again indicating that the ratio of the axons is negatively correlated with the rate of synapse elimination (S4a). These results and those reported above demonstrate that the model reliably predicts a close correlation between the rate of synapse elimination and the ratio of the contact areas formed by axons, tSCs, and vacancies consistent with our previous results and studies[1-3]. They also support the direct involvement of vacancies in synapse elimination. However, the seemingly positive correlation of the rate of synapse elimination with the relative area of tSCs and that of vacancies need to be interpreted with caution because the relative areas of tSCs, vacancies, and axons are inter-dependent. By using the composite area defined as the product of the relative area of tSCs (A_{tSC}) and that of vacancies (A_{vac}) divided by the relative area of axons (A_{Axon}), we found that the composite area is negatively correlated with the number of iterations to complete synapse elimination (Spearman Correlation, $p<0.05$) as shown in S4b.

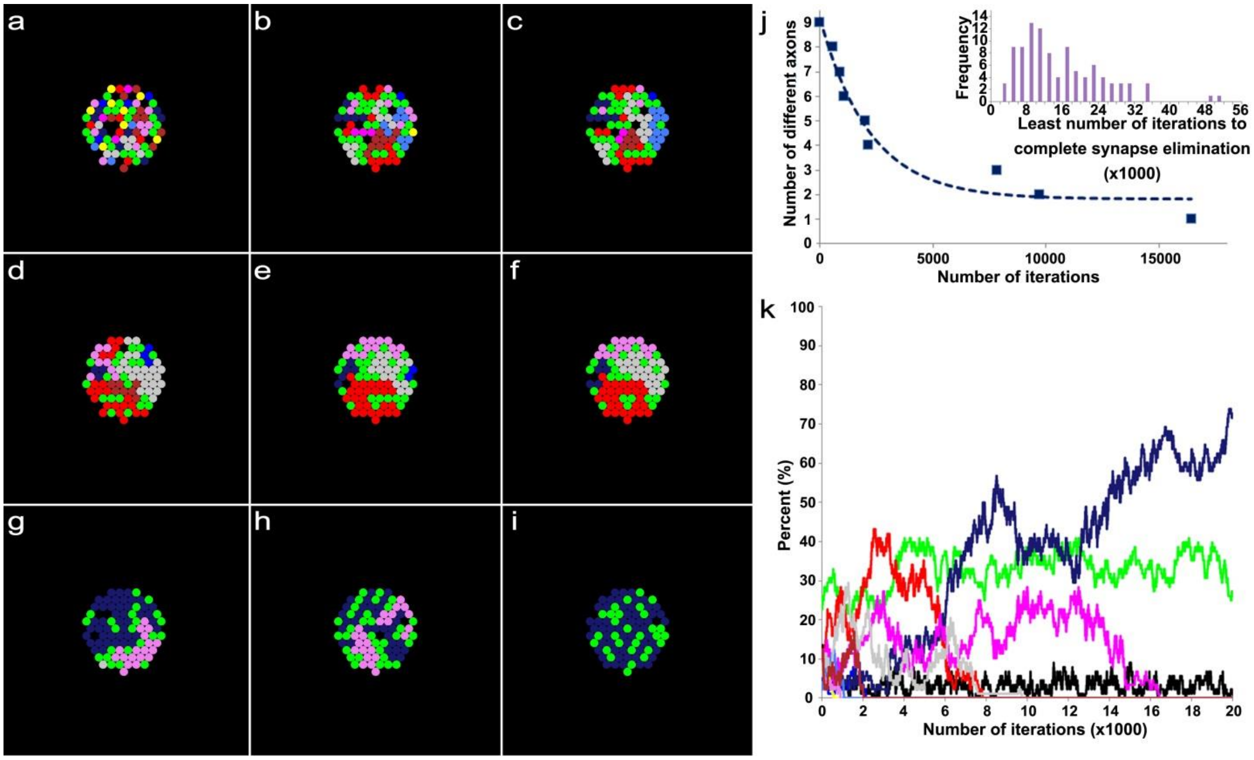

S5. An example result based on a model of synaptic competition among axons, tSCs, and vacancies with different constant transition probabilities derived from the area ratios at P7 of the NMJs. Simulations carried out using the same configuration as described in Fig. 3, but the final ratios of tSCs, vacancies and axons are set to be 0.55, 0.05, and 0.40, respectively, which are the ratios at P7 of the NMJs (See Methods). (a) Initially, 9 different axons, tSCs and vacancies form their contact sites randomly on a muscle fiber with the initial ratio of their total contact areas (axons:tSCs:vacancies), which is about 30:16:54 as S1, S2, S3, and Fig. 3. (b-h) The competition among axons, tSCs, and vacancies with their random transition probabilities shows elimination of multiple contact sites formed by different axons, tSCs, and vacancies at different iterations of the simulation (600, 900, 1100, 2100, 2200, 7900, and 9800 iterations, respectively). (i) When the simulation is at 16,500 iterations, the competition leads to a complete synapse elimination with tSC and vacant sites present demonstrating that optimal transition probabilities reliably simulate synapse elimination. (j) The number of different types of axons in the endplate reduces sharply as the iteration proceeds. Inset: a distribution of the least number of iterations to complete synapse elimination for 100 repeated simulations (15730 ± 943, Mean ± SE). (k) Change of ratios of the contact areas formed by tSCs (green), vacancies (black), and 9 different axons (colors different from green and black) as the simulation based on the stochastic model of tSC and vacancy mediated synapse elimination proceeds. Inset: the distribution of the least number of iterations when synapse elimination is complete from 100 repeated simulations.

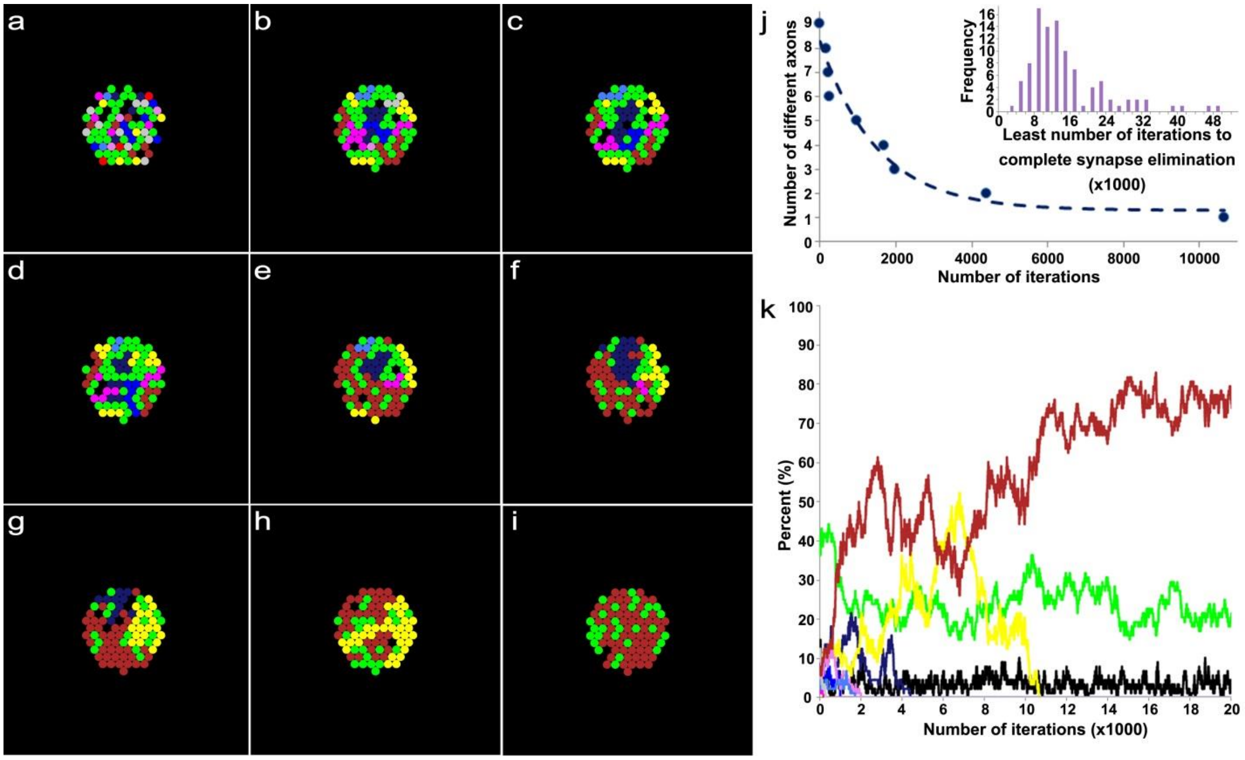

S6. An example result based on a model of synaptic competition among axons, tSCs, and vacancies with different constant transition probabilities derived from the area ratios at P16 of the NMJs. Simulations carried out using the same configuration as described in Fig. 3, but the final ratios of tSCs, vacancies and axons are set to be 0.41, 0.06, and 0.53, respectively, which are the ratios at P16 of the NMJs (See Methods). (a) Initially, 9 different axons, tSCs and vacancies form their contact sites randomly on a muscle fiber with the initial ratio of their total contact areas (axons:tSCs:vacancies), which is about 30:16:54 as S1, S2, S3, S5, and Fig. 3. (b-h) The competition among axons, tSCs, and vacancies with their random transition probabilities shows elimination of multiple contact sites formed by different axons, tSCs, and vacancies at different iterations of the simulation (200, 300, 400, 1000, 1700, 2000, and 4400 iterations, respectively). (i) When the simulation is at 10,600 iterations, the competition leads to a complete synapse elimination with tSC and vacant sites present demonstrating that optimal transition probabilities reliably simulate synapse elimination. (j) The number of different types of axons in the endplate reduces sharply as the iteration proceeds. Inset: a distribution of the least number of iterations to complete synapse elimination for 100 repeated simulations (15016 ± 902, Mean ± SE). (k) Change of ratios of the contact areas formed by tSCs (green), vacancies (black), and 9 different axons (colors different from green and black) as the simulation based on the stochastic model of tSC and vacancy mediated synapse elimination proceeds.

References

1 Lee, Y. I. *et al.* Neuregulin1 displayed on motor axons regulates terminal Schwann cell-mediated synapse elimination at developing neuromuscular junctions. *Proc Natl Acad Sci U S A* **113**, E479-487, doi:10.1073/pnas.1519156113 (2016).

2 Smith, I. W., Mikesh, M., Lee, Y. & Thompson, W. J. Terminal Schwann cells participate in the competition underlying neuromuscular synapse elimination. *J Neurosci* **33**, 17724-17736, doi:10.1523/JNEUROSCI.3339-13.2013 (2013).

3 Lee, Y. I., Mikesh, M., Smith, I., Rimer, M. & Thompson, W. Muscles in a mouse model of spinal muscular atrophy show profound defects in neuromuscular development even in the absence of failure in neuromuscular transmission or loss of motor neurons. *Dev Biol* **356**, 432-444, doi:10.1016/j.ydbio.2011.05.667 (2011).
